# Supplementary material for: From sequence to enzyme mechanism using multi-label machine learning
Source: BMC Bioinformatics. 2014 May 19;15:150. doi: 10.1186/1471-2105-15-150 (PMC4229970; doi:10.1186/1471-2105-15-150)
Supplement: Additional file 2 — Java code of ml2db. Additional file ml2db_code.tar.gz contains the Java source code to run the multi-label machine learning experiments and save the results to database. The code’s Javadoc is included. [file 1471-2105-15-150-S2.zip › additional file 2/ml2db/ml2db/doc/test/mulan/measuremetrics/AnnotationsReaderTest.html]

AnnotationsReaderTest


---


|  |  |  |
| --- | --- | --- |
| **Overview** \| **Package** \| **Class** \| **Use** \| **Tree** \| **Deprecated** \| **Index** \| **Help** | |  |
| PREV CLASS   **NEXT CLASS** | **FRAMES**    **NO FRAMES**     **All Classes** |  |
| SUMMARY: NESTED \| FIELD \| CONSTR \| METHOD | DETAIL: FIELD \| CONSTR \| METHOD |  |


---


## test.mulan.measuremetrics Class AnnotationsReaderTest

```
java.lang.Object
  junit.framework.Assert
      junit.framework.TestCase
          test.mulan.measuremetrics.AnnotationsReaderTest
```

**All Implemented Interfaces:**: junit.framework.Test

---

``` public class AnnotationsReaderTest extends junit.framework.TestCase ```

Class

**Version:**
:   5 Dec 2011

**Author:**
:   Luna De Ferrari luna.deferrari@ed.ac.uk or
    luna.deferrari@st-andrews.ac.uk

---

| **Field Summary** | |
| --- | --- |
| `static java.lang.String` | `instances1` |
| `static java.lang.String` | `path` |
| `static java.lang.String` | `pred1` |
| `static java.lang.String` | `truth1` |


**Constructor Summary**

`AnnotationsReaderTest()`


| **Method Summary** | |
| --- | --- |
| `static AnnotationsReader` | `getAnnotationsReader()`             Test method for `uk.ac.ed.inf.enzml.mulan.measuremetrics.AnnotationsReader#AnnotationsReader(java.lang.String, java.lang.String, java.lang.String)` . |
| `void` | `testGetBipartitions()`             Test method for `AnnotationsReader.getPredictedBipartitions()` . |
| `void` | `testGetInstances()`             Test method for `AnnotationsReader.getInstances()` . |
| `void` | `testGetInstancesAndLabels()`             Test method for `AnnotationsReader.getPredictionsInstancesAndLabels()` . |
| `void` | `testGetLabels()`             Test method for `AnnotationsReader.getLabels()` . |
| `void` | `testGetMLOutput()` |

**Methods inherited from class junit.framework.TestCase**

`countTestCases, getName, run, run, runBare, setName, toString`

**Methods inherited from class junit.framework.Assert**

`assertEquals, assertEquals, assertEquals, assertEquals, assertEquals, assertEquals, assertEquals, assertEquals, assertEquals, assertEquals, assertEquals, assertEquals, assertEquals, assertEquals, assertEquals, assertEquals, assertEquals, assertEquals, assertEquals, assertEquals, assertFalse, assertFalse, assertNotNull, assertNotNull, assertNotSame, assertNotSame, assertNull, assertNull, assertSame, assertSame, assertTrue, assertTrue, fail, fail, failNotEquals, failNotSame, failSame, format`

**Methods inherited from class java.lang.Object**

`equals, getClass, hashCode, notify, notifyAll, wait, wait, wait`

**Field Detail**

### path

```
public static java.lang.String path
```

---


### instances1

```
public static java.lang.String instances1
```

---


### pred1

```
public static java.lang.String pred1
```

---


### truth1

```
public static java.lang.String truth1
```


**Constructor Detail**

### AnnotationsReaderTest

```
public AnnotationsReaderTest()
```


**Method Detail**

### testGetBipartitions

```
public void testGetBipartitions()
```

:   Test method for
    `AnnotationsReader.getPredictedBipartitions()`
    .

---


### testGetInstances

```
public void testGetInstances()
```

:   Test method for
    `AnnotationsReader.getInstances()`
    .

---


### testGetInstancesAndLabels

```
public void testGetInstancesAndLabels()
```

:   Test method for
    `AnnotationsReader.getPredictionsInstancesAndLabels()`
    .

---


### testGetLabels

```
public void testGetLabels()
```

:   Test method for
    `AnnotationsReader.getLabels()`
    .

---


### testGetMLOutput

```
public void testGetMLOutput()
```

---


### getAnnotationsReader

```
public static AnnotationsReader getAnnotationsReader()
```

:   Test method for
    `uk.ac.ed.inf.enzml.mulan.measuremetrics.AnnotationsReader#AnnotationsReader(java.lang.String, java.lang.String, java.lang.String)`
    .


---


|  |  |  |
| --- | --- | --- |
| **Overview** \| **Package** \| **Class** \| **Use** \| **Tree** \| **Deprecated** \| **Index** \| **Help** | |  |
| PREV CLASS   **NEXT CLASS** | **FRAMES**    **NO FRAMES**     **All Classes** |  |
| SUMMARY: NESTED \| FIELD \| CONSTR \| METHOD | DETAIL: FIELD \| CONSTR \| METHOD |  |


---
